# Creation of Superhydrophobic Poly(L-phenylalanine) Nonwovens by Electrospinning

**DOI:** 10.3390/polym10111212

**Published:** 2018-10-31

**Authors:** Hiroaki Yoshida, Kazuhiro Yanagisawa

**Affiliations:** Faculty of Textile Science and Technology, Shinshu University, 3-15-1 Tokida, Ueda, Nagano 386-8567, Japan; 17FS424E@shinshu-u.ac.jp

**Keywords:** superhydrophobicity, petal effect, poly(L-phenylalanine), electrospinning

## Abstract

From the viewpoint of green chemistry and environmental chemistry, an important challenge in the field of superhydrophobic materials is to create them with only bio-based molecules. We developed superhydrophobic and chemically stable poly(L-phenylalanine) (PolyPhe) nonwovens by electrospinning. PolyPhe was selected because, due to its very rigid chemical structure, it is one of the toughest and most hydrophobic polymers among polymers composed only of amino acids. The water contact angle on the nonwovens is a maximum of 160°, and the droplets are stably adhered and remain still on the nonwoven surface even if it is turned over, thereby suggesting a petal-type superhydrophobicity. The nonwovens show a good chemical stability, and their weight remains unchanged after 5 days immersion in acidic (pH 2) and basic (pH 12) conditions. In addition, the superhydrophobic property is not lost even after the alkali treatment. Such tough superhydrophobic materials are intriguing for further biomedical and environmental applications.

## 1. Introduction

Over the last few decades, a variety of superhydrophobic materials have been artificially designed and developed, bioinspired by fascinating superhydrophobic phenomena in nature such as the rolling of water droplets on a lotus leaf (known as a lotus effect) and the adhesion of water droplets on a rose petal (a petal effect) [1,2,3,4,5]. To date, it has been clarified that two important factors—use of chemicals showing low surface free energy (e.g., alkyl, fluoroalkyl chains, etc.) and introduction of rough or topological features on their surfaces—are indispensable for the creation of superhydrophobic materials. As a result, superhydrophobic properties can be now imparted to the surfaces of various organic, polymeric, and inorganic components, simply by modifying fluorinated compounds on a rough material surface. However, considered almost all such materials are non-degradable, there is an undoubted risk of using fluorinated compounds [6], making them unable to be used for biomedical and green/environmental applications. Because of these factors, a safe and secure design of superhydrophobic materials is strongly required. As far as we know, there are three reports on superhydrophobic materials composed of only biodegradable polymers. Osawa et al. and Mano et al. reported the construction of lotus-type superhydrophobic films composed of poly(ε-caprolactone) [7] and poly(L-lactic acid) [8,9], respectively, and both films showed poor bacteria adhesion. More recently, we reported that electrospinning of poly(γ-glutamic acid) (γ-PGA) modified with L-phenylalanine ethylester (γ-PGA-Phe) gave petal-type superhydrophobic materials based on hydrophobic benzene rings contained in Phe and rough fiber structures [10]. Interestingly, the nonwovens were biodegraded in alkaline conditions and allowed good adhesion/proliferation for various cells. Such kinds of non-fluorinated, superhydrophobic and biodegradable materials are intriguing for further biomedical and environmental applications. However, plausible bio-related components (e.g., polyesters, polypeptides, polysaccharides, etc.) are too wide. Thus, a further possibility for amino acid-based polymers for creation of superhydrophobic materials is discussed below.

It is interesting to consider what the most hydrophobic polymer composed only of amino acids actually is. In simplistic terms, because Phe has the highest value in terms of hydrophobicity index of -amino acid [11], one of the highest hydrophobicity out of poly(amino acid)s is expected to be a Phe homopolymer. Herein, we focus on electrospinning of polyphenylalanine (PolyPhe) for development of new biodegradable, superhydrophobic nonwoven materials. The synthesis of PolyPhe began with *N*-carboxyanhydride (NCA) polymerization in the late 1950s [12,13]; however, there are very few works on the material processing of PolyPhe because of its poor solubility in both aqueous and organic environments [14,15]. Although Kamei reported that PolyPhe with molecular weight of approximately 1900 (13 mer) can be dissolved in CHCl_3_ [15], it would be desirable to use higher-molecular-weight PolyPhe to suppress the effect of functional groups (carboxyl and amine groups) at the polymer end. To process PolyPhe into various-shaped materials, an appropriate solvent condition for higher molecular weight PolyPhe must be explored. The synthesis of PolyPhe was performed by polycondensation with highly activated esters of amino acids, as previously reported by Kamei et al. [15]. This method was chosen as a promising route for producing poly(amino acid)s without having to use dangerous reagents such as phosgene or triphosgene. Another advantage of PolyPhe for superhydrophobic materials is good stability in both acidic and basic conditions. Matsusaki et al. reported that PolyPhe were not degraded, even after immersion in 5 M of NaOH for 3 weeks [16]. This property would be important for construction of tough superhydrophobic materials stable in various conditions. Electrospinning is a facile and versatile technique for preparing nano- to micro-sized fibers from various materials, such as polymers, composites and sol-gels, and the resultant fibrous structure drastically increases surface roughness of the materials [17,18,19,20,21]. Furthermore, contacting of polymer solutions with the air at much larger surface area during electrospinning would be expected to accumulate benzene rings on the surface of the forming fibers, enhancing the surface hydrophobicity, while Phe units are often used in the self-assembly process for preparation of various functional materials [22,23,24]. It is also meaningful to succeed in PolyPhe electrospinning because there are only a few reports on electrospinning of poly(amino acid)s such as γ-PGA [25], polyornithine [26], Poly(γ-benzyl α, L-glutamate) [27], and synthetic anionic copolypeptide of L-glutamic acid and L-tyrosine [28]. Taken together, electrospinning of higher-molecular-weight PolyPhe would produce a novel superhydrophobic and biodegradable materials composed solely of amino acids.

## 2. Materials and Methods

### 2.1. Synthesis and Characterization of PolyPhe and Investigation of Its Solubility in Various Solvents

PolyPhe was synthesized according to the previous method [15]. Briefly, 8.3 g (50 mmol) of Phe (Nacalai Tesque Inc., Kyoto, Japan) was reacted with 10.1 g (50 mmol) of 4-nitrophenyl chloroformate (Combi-Blocks Inc., San Diego, CA, USA) in 120 mL of ethyl acetate (Nacalai Tesque Inc., Kyoto, Japan) at 45 °C for 24 h under argon atmosphere to produce the activated esters of Phe in 73% yield. 0.33 g (1.0 mmol) of the esterified Phe was then polycondensated in 0.5 mL of dimethylacetamide (Nacalai Tesque Inc., Kyoto, Japan) at 60 °C for 1 week to obtain the target PolyPhe in 35% yield. The product was characterized by nuclear magnetic resonance (NMR) (Magnet System 400’54 Ascend, Bruker, Billerica, MA, USA) and FT-IR (FT/IR-4700ST, JASCO, Tokyo, Japan). The average molecular weight of the synthesized polymer was determined in 10 mmol/L sodium trifluoroacetate in hexafluoroisopropanol (HFIP) by gel permeation chromatography (GPC) (Shodex GPC LF-404), calibrated with poly(methyl methacrylate) standards at a flow rate of 0.3 mL/min at 40 °C. The weight-average molecular weight (*M*_w_) was 3900 and the molecular-weight distribution (*M*_w_/*M*_n_) was 1.3.

Solubility of PolyPhe was investigated with trifluoroacetic acid (TFA) (Nacalai Tesque Inc., Kyoto, Japan), chloroform (CHCl_3_) (Nacalai Tesque Inc., Kyoto, Japan), HFIP (99.5%, Oakwood Products, Inc., Estill, SC, USA), and their mixtures at 25–60 °C.

### 2.2. Electrospinning of PolyPhe in the Air and in the Nonpolar Solvents

A Nanofiber Electrospinning Unit (Kato Tech Co., Ltd., Kyoto, Japan) was used for electrospinning in the air. The 32 *w*/*v*% of PolyPhe in the mixed solvents of TFA/CHCl_3_ (9/1 *v*/*v*) was pumped through a single-use blunt-end 18-gauge cannula at a flow rate of 0.5–1.0 mL/h, and the collection distance between the cannula and aluminum foil target was 10 cm. A voltage of 30 kV was applied between the cannula and the substrate.

Electrospinning in the nonpolar solvents was examined according to the new method developed by Wakisaka and Tsuchiya [29,30]. Briefly, polymer solutions were pumped at the speed of 0.5 mL/h from the tip of the stainless-steel needle immersed in the nonpolar solvents (e.g., hexane, cyclohexane, etc.) poured in a 200 mL beaker. The solution jet, starting from the tip, went to the surface of a stainless-steel mesh coated with aluminum foil set on the bottom of the beaker. The voltage of 5 kV was applied between the needle and the mesh. Collection distance was fixed at 3 cm.

Morphologies of PolyPhe constructs deposited on the aluminum substrates were observed by 3D measuring laser microscope (LM) (LEXT OLS4100, OLYMPUS, Tokyo, Japan) or SEM (JSM-6010LA, JEOL Ltd., Tokyo, Japan). Fiber diameter, length, and aspect ratio were calculated from SEM images (*n* = 50). FT-IR and XRD (MiniFlex 300, Rigaku, Tokyo, Japan) measurements were also done for characterization of PolyPhe constructs. Root-mean square (RMS) surface roughness of the obtained nonwovens was calculated by the LM.

### 2.3. Water Contact Angle (CA) Measurement on the Surface of PolyPhe Nonwovens

CA was measured by DropMaster300 Kyowa Interface Co., Ltd., Saitama, Japan. A 2 µL of water droplet was put on the collected PolyPhe nonwovens and temporal change of CA was measured every 30 s over 5 min. To examine the effect of the water droplet pH on CA, 0.01 M hydrochloric acid (HCl) aq. and 0.01 M sodium hydroxide (NaOH) aq. were used.

### 2.4. Hydrolysis Test of PolyPhe Nonwovens under Acidic, Basic, Enzymatic Conditions

Approximately 3 mg of the PolyPhe nonwovens were forcibly immersed into 0.01 M HCl aq., 5 M HCl aq., 0.01 M NaOH aq., 5 M NaOH aq., and 0.4 *w*/*v*% pepsin (Nacalai Tesque Inc., Kyoto, Japan) in 0.01 HCl aq. for 1 or 5 day. Morphological changes of the nonwovens at the prescribed degradation times were observed with the LM.

## 3. Results and Discussion

### 3.1. Solubility of PolyPhe in Various Solvents

Based on the results of extensive solubility tests, it was found that TFA/CHCl_3_ (9/1 *v*/*v*) could dissolve the high-molecular-weight PolyPhe at concentrations of up to 32 *w*/*v*%. HFIP also dissolved it at 40 °C. Unless otherwise stated, in the following section, TFA/CHCl_3_ (9/1 *v*/*v*) is used as a solvent for PolyPhe.

### 3.2. Electrospinning for the Preparation of PolyPhe Nonwovens

The synthesized PolyPhe was dissolved in TFA/CHCl_3_ (9/1 *v*/*v*) at a concentration of 32 *w*/*v*%. The solution was then electrospun in the air, but no fiber formation was observed (Figure S1), possibly because of low polymer concentration and low solution viscosity. Thus, as an alternative approach, we focused on a novel electrospinning technique in the nonpolar solvents developed by Wakisaka and Tsuchiya [29,30]. Different from conventional electrospinning in the air and wet electrospinning (spinning in the air and fiber collection in the bath) [31,32,33], all processes including spinning with this method are done in a non-polar, electrically insulating solvent (such as hexane, cyclohexane, etc.). They also mentioned that using this technique the instability of Taylor cone often observed during electrospinning in the air can be efficiently suppressed, and that electrospinnability can be tuned by the surrounding conditions [30]. In addition, because a nonpolar solvent used in the bath for fiber collection is indeed a poor solvent for the polymer, polymer precipitation should occur throughout solvent exchange, which may induce crystallization of the polymer. Furthermore, fiber formation in a nonpolar solvent is likely to effectively accumulate benzene rings on the surface of the forming fibers for enhancing surface hydrophobicity of the resultant nonwovens.

**Figure 1:**
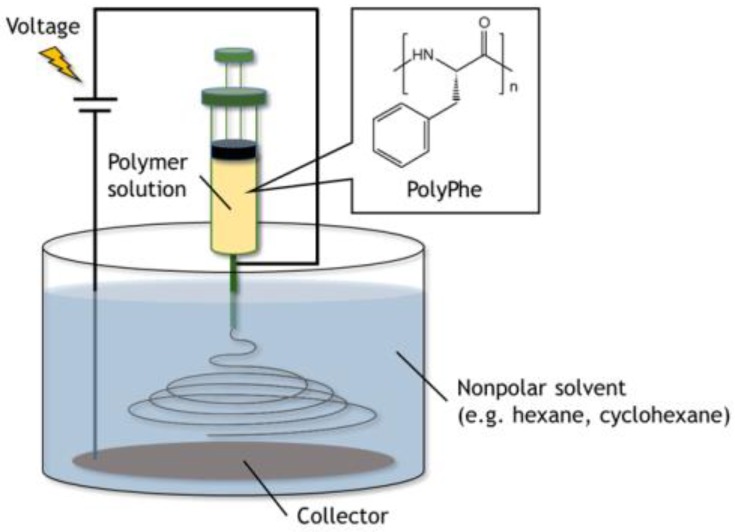
The experimental setup for electrospinning used in this study. All processes including spinning were done in a nonpolar solvent.

**Figure 2:**
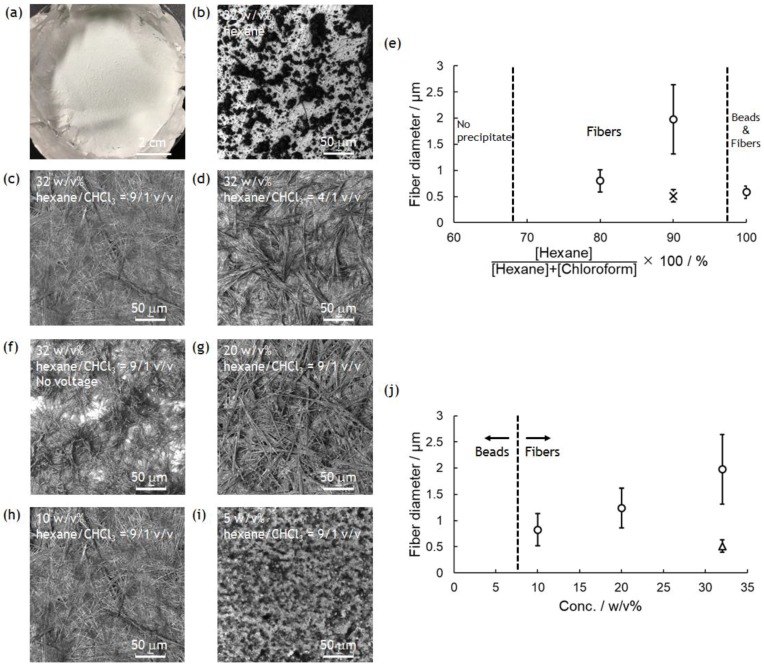
Electrospinning of PolyPhe in TFA/CHCl_3_ (9/1 *v*/*v*) into hexane/CHCl_3_ (10/0, 9/1, 4/1 *v*/*v*). PolyPhe conc. was 32, 20, 10, or 5 *w*/*v*%. (**a**) A typical photograph of PolyPhe nonwovens collected on the aluminum foil. (**b**–**d**,**f**–**i**) LM images of the electrospun PolyPhe constructs. The voltage was (**b**–**d**,**g**–**i**) 5 kV and (**f**) 0 kV, solution speed was 0.5 mL/h, and collection distance was 3 cm. (**e**) Solvent composition vs fiber diameter. PolyPhe conc. was fixed at 32 *w*/*v*%. (**j**) PolyPhe conc. vs fiber diameter. The solvent was hexane/CHCl_3_ (9/1). The diameter was calculated from SEM images of three different samples (*n* = 50). A cross in (**e**) is the value calculated from the image (**f**). A triangle in (**j**) is the value calculated from the cast film.

The experimental device was set up as shown in Figure 1. As the PolyPhe solution was electrospun into hexane, white precipitate formed too quickly onto the aluminum substrate (Figure 2a). LM image of the obtained precipitate showed the coexistence of fibers as a minor product and beads as a major product, possibly because of the overly rapid solvent exchange (Figure 2b). Thus, it would be important to delay such a rapid precipitation of PolyPhe in the hexane bath. An easy approach is the addition of CHCl_3_ in hexane, which is expected to give better mixing properties with polymer solution. It was indeed confirmed that the increased amount of CHCl_3_ clearly decreased the amount of PolyPhe precipitation in the bath. As the solvent composition reached hexane/CHCl_3_ (9/1 *v*/*v*), the electrospinning became stable over 1 h, and the resultant constructs were only homogeneous, rod-like fibers (Figure 2c). Then, similar but thinner and shorter fibers were obtained at hexane/CHCl_3_ (4/1 *v*/*v*) (Figure 2d), only beads were observed at hexane/CHCl_3_ (2/1 *v*/*v*), and no precipitate was observed at hexane/CHCl_3_ (1/1 *v*/*v*), because solution properties of polymer solution and bath might be similar. The relationship of fiber diameter/length and solvent composition of the bath supports the formation of thinner and shorter fibers with smaller aspect ratios as the volume percentage of CHCl_3_ increases; that is, as two solvent properties became closer (Figure 2e and Figure S2a,b). We also examined whether applied voltage is needed for the formation of PolyPhe fibers, because this electrospinning process should include simple precipitation of PolyPhe in poor solvents such as hexane or hexane/CHCl_3_. When 32 *w*/*v*% PolyPhe solution was extruded from the tip of the needle into hexane/CHCl_3_ (9/1 *v*/*v*) under no voltage, shorter and thinner rods were formed (Figure 2f), suggesting that the electrospinning process made a good contribution to the formation of long PolyPhe fibers. Next, the effect of PolyPhe concentration on fiber diameter was also investigated with hexane/CHCl_3_ (9/1 *v*/*v*) as a bath. As the concentration went down to 20 and 10 *w*/*v*%, the average diameter/length of the obtained fibers decreased to 1.2/40 µm and 0.8/19 µm (Figure 2g–j and Figure S2c,d). This result shows a similar tendency to that of conventional electrospinning that fiber diameter increases as polymer concentration increases. As shown in Figure S3a, PolyPhe fiber fabrication by electrospinning worked well with cyclohexane instead of hexane, and the diameter of the formed fibers was thinner than that in hexane (Figure 2c). Although the cause was not fully understood, it might be due to the slight difference of solvent properties between hexane and cyclohexane. Overall, we succeeded in electrospinning of PolyPhe into the nonpolar solvents, and the fiber diameter could be controlled by the solvent composition of the bath and polymer concentration.

**Figure 3:**
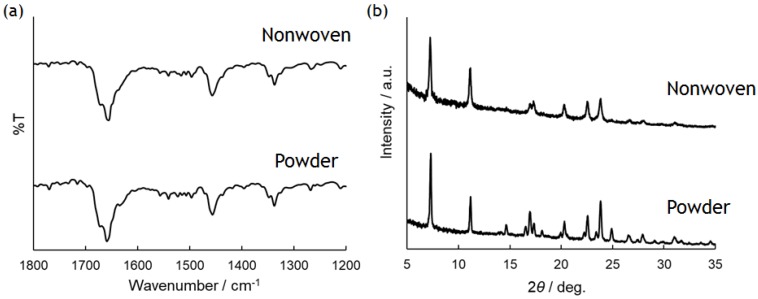
(**a**) FT-IR spectra and (**b**) XRD patterns of as-synthesized and nonwoven PolyPhe prepared by electrospinning of 32 *w*/*v*% solution.

Characterization of the obtained PolyPhe fibers was performed by FT-IR and XRD measurements with the sample prepared by electrospinning of 32 *w*/*v*% PolyPhe solution into hexane/CHCl_3_ (9/1 *v*/*v*) (Figure 2c) as a representative. FT-IR spectra did not show any difference between the as-synthesized polymers and the obtained fibers, suggesting that the chemical structure of the polymers in the fibers was close to the original structures (Figure 3a). Furthermore, the peak at about 1650 cm^−1^ indicates that both PolyPhe fibers contain α-helix structures [34]. As shown in Figure 2, PolyPhe fibers were rod-like, rigid structures, and it is therefore interesting to examine the crystalline properties of their fibers by XRD measurement. As a result, the sharp peak pattern of PolyPhe fibers was quite similar to that of the as-synthesized PolyPhe powders, suggesting the formation of crystalline PolyPhe fibers (Figure 3b). For a comparison, the XRD results of the fibers prepared by electrospinning of PolyPhe into the air, which was synthesized by polymerization of Phe-NCA in the presence of triphosgene and trimethylamine, showed amorphous characteristics, demonstrating that electrospinning into a nonpolar solvent would effectively induce the crystallization of PolyPhe (Figure S4). These results were also supported by the difference in the association behavior of diphenylalanine, that is, H-aggregate or J-aggregate [24].

### 3.3. Wettability of PolyPhe Nonwovens

**Figure 4:**
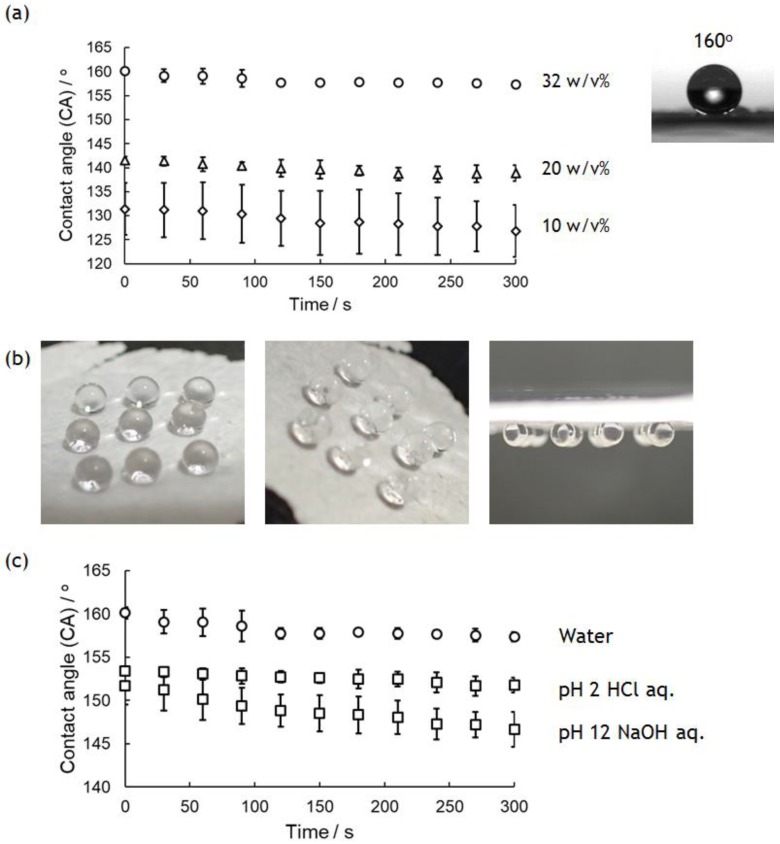
Wettability of PolyPhe nonwovens prepared by electrospinning into hexane/CHCl_3_ (9/1 *v*/*v*). (**a**) CA change on PolyPhe nonwovens electrospun at different polymer concentrations (*n* = 3). (**b**) Strong adhesion of water droplet onto PolyPhe nonwovens with a tilt angle of 0, 45, and 90°. (**c**) CA change of acidic (pH 2) and basic (pH 12) droplets on the nonwovens (32 *w*/*v*%) (*n* = 3).

Surface wettability of PolyPhe nonwovens was investigated by evaluating the CA of a 2 μL water droplet onto a nonwoven fixed onto a glass plate over 5 min. Figure 4a shows the CA change of water droplets on the nonwovens prepared by electrospinning of 32, 20, and 10 *w*/*v*% PolyPhe solutions into hexane/CHCl_3_ (9/1 *v*/*v*). The initial value of the CA increased with increasing polymer concentrations used, and it is surprising that the value of 32 *w*/*v*% PolyPhe nonwovens was 160°, suggesting superhydrophobicity. These hydrophobic properties were stable, and there was almost no change over 5 min. Water droplets on the nonwovens were stably adhered and stayed still on the nonwoven surface even if it was turned over (Figure 4b). This is known as a petal-type superhydrophobic surface, and it has often been observed on surfaces containing randomly arranged needles and fibers because of the presence of less or no air between the material surface and the water droplets [35,36,37]. Furthermore, the cast-film was prepared by air-drying PolyPhe solution on a glass plate as a control. The CA of water droplets on the film was only about 80°, much smaller than those on the nonwovens (Figure S5). It is interesting to consider that LM image of the film showed similar fiber structures (diameter: 0.5 ± 0.1 µm) to those of the electrospun materials. Considering the relationship of the apparent contact angle on a rough surface and the Young contact angle on a flat surface given by Wenzel’s equation [38], the CA values on the nonwovens (160°) and cast-films (80°) prepared from PolyPhe may seem strange. Yarin et al. reported a series of the convincing results on such hydrophobicity on electrospun nonwovens and concluded that the static CA value on nanofiber mat is more influenced by surface structures of the materials rather than their chemical structures [39,40,41], supporting our results that PolyPhe nonwovens showed a large CA value compared to that of the corresponding cast-film. As stated above, higher polymer concentration gave thicker fibers, and thus, thicker fibers should result in the formation of a surface with greater roughness. Surface root-mean square (RMS) roughness of the nonwovens analyzed with the laser microscope supported our conclusion that the nonwovens composed of thicker fibers produced rougher surface (Figure S6). The RMS roughness of the cast-film was much lower than those of the nonwovens, resulting in the least hydrophobicity. In addition to fiber diameter, the kind and composition of the solvent also influence the hydrophobicity of the nonwoven surface. The fibers prepared by using cyclohexane also decreased the CA value slightly, possibly because of the formation of thinner fibers (Figure S3b). When the composition of the bath was changed from hexane/CHCl_3_ (9/1 *v*/*v*) to hexane/CHCl_3_ (4/1 *v*/*v*), the CA drastically decreased from 160° to 94° (Figure S7a). This value is too small compared to the CA (131°) of 10 *w*/*v*% nonwovens with similar fiber diameter, which may be caused by the decreased number of benzene rings presenting on the nonwoven surface as the amount of CHCl_3_ increased. As shown in Figure 2f, the construct prepared under no voltage also showed a high CA value on its surface (154°); however, time-dependent wetting was observed (Figure S7b). Shortening of the fibers might have increased the number of fiber ends with ionic functional groups. The wettability change under both acidic (pH 2) and basic (pH 12) conditions was also monitored, because PolyPhe has good stability in such solutions (Figure 4c). The nonwovens still kept their superhydrophobicity at pH 2, but were slightly below 150° at pH 12. Although details are unclear, the ionization of functional groups at the fiber ends would be influential. Taken together, these results suggest that the surface hydrophobicity of PolyPhe materials can be tuned from less hydrophobic to superhydrophobic by controlling the fiber thickness and length, the density of benzene rings on their surface, and surface roughness of the materials.

### 3.4. Chemical Stability of PolyPhe Nonwovens

**Figure 5:**
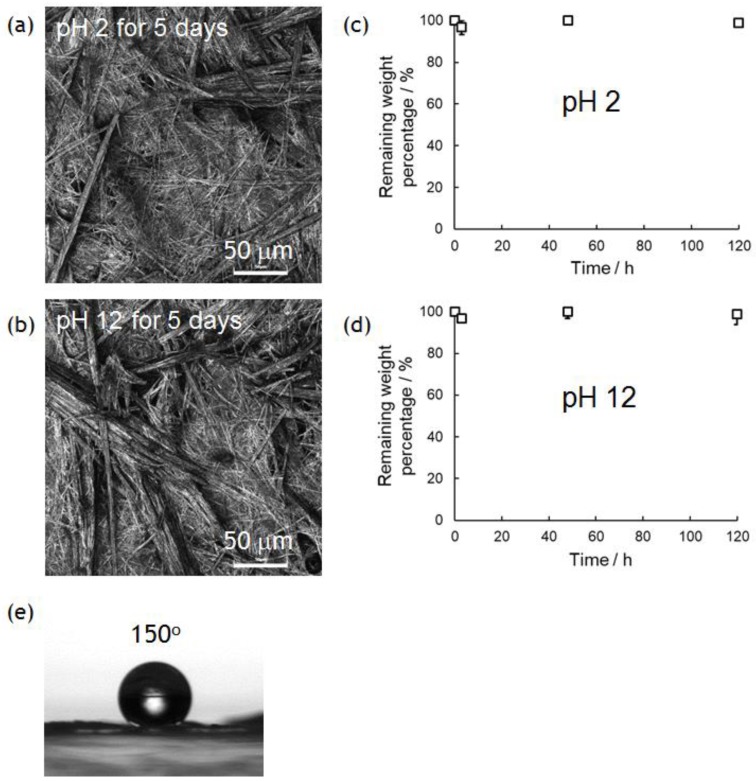
Chemical stability of PolyPhe nonwovens prepared by electrospinning into hexane/CHCl_3_ (9/1 *v*/*v*) in acidic (pH 2) and basic (pH 12) conditions. (**a**,**b**) Weight remaining (*n* = 3) and (**c**,**d**) LM images of the PolyPhe nonwovens after 5 days’ immersion in (**a**,**c**) 0.01 M HCl aq. and (**b**,**d**) 0.01 M NaOH aq. (**e**) The CA of a water droplet on the PolyPhe nonwovens after the alkali treatment.

In previous research, we demonstrated the hydrolytic property of superhydrophobic materials composed of only amino acids [10]. This would be quite important in developing truly biomimetic/bioinspired materials for biomedical and green/environmental applications, because conventional superhydrophobic materials containing non-degradable parts (typically fluorinated compounds) are difficult to hydrolyze in similar conditions. PolyPhe with rigid main chains used in this study is expected to be much tougher than previous polymers with flexible main chains. Thus, the nonwovens prepared by electrospinning should show better chemical stability in both acidic and basic solutions. Approximately 3 mg of the PolyPhe nonwovens prepared by electrospinning into hexane/CHCl_3_ (9/1 *v*/*v*) were forcibly immersed in 0.01 M HCl aq. (pH 2) or 0.01 M NaOH aq. solution (pH 12) at ambient temperature. Interestingly, the weight of the nonwovens was not changed at all in both conditions even after 5 days (Figure 5a,b) and the morphologies of the nonwoven surfaces were almost the same as those before the treatments (Figure 5c,d). FT-IR spectra indicated no significant damage by the treatments (Figure S8). Similar hydrolytic experiments in 5 M HCl aq. at 80 °C for 24 h and 5 M NaOH aq. at 80 °C for 24 h were done in the same manner, but no weight change was observed. Enzymatic degradation was also tried with pepsin, which efficiently cleaves peptide bonds between aromatic amino acids [42], but no change occurred. Furthermore, the superhydrophobic property was also re-tested with the nonwovens after 5 days’ immersion in a 0.01 M NaOH aq. at ambient temperature. Surprisingly, the water CA on the nonwovens was still over 150° (Figure 5e). Such results are enough to demonstrate chemical stability of the PolyPhe nonwovens.

## 4. Conclusions

In conclusion, we successfully prepared petal-type superhydrophobic nonwovens with great chemical stability. The nonwovens could be prepared by electrospinning of PolyPhe into a non-polar solvent. The hydrophobic property was tuned from less hydrophobic to superhydrophobic by controlling the surface roughness and the number of benzene rings on the fiber surface. The nonwovens were chemically stable even after acidic and basic treatments for a few days, and the operated materials still retained their superhydrophobicity. We believe this material is the toughest and most hydrophobic among materials designed with only amino acids, and that such superhydrophobic materials are ideal for biomedical and environmental applications.

## Supplementary Materials

The following (Figures S1–S8) are available online at http://www.mdpi.com/2073-4360/10/11/1212/s1.
