# Supplementary material for: Creation of Superhydrophobic Poly(L-phenylalanine) Nonwovens by Electrospinning
Source: Polymers (Basel). 2018 Oct 31;10(11):1212. doi: 10.3390/polym10111212 (PMC6290602; doi:10.3390/polym10111212)
Supplement: Supplementary file 1 [file polymers-10-01212-s001.pdf]

Supplementary Materials: Creation of Superhydrophobic Poly(L-Phenylalanine) Nonwovens by Electrospinning

Hiroaki Yoshida and Kazuhiro Yanagisawa

Contents

1. (Figure S1) Electrospinning of PolyPhe into the air S2
2. (Figure S2) Investigations on length and aspect ratio of the obtained fibers S2
3. (Figure S3) Electrospinning of PolyPhe into cyclohexane/$CHCl_3$ (9/1 v/v) S3
4. (Figure S4) Electrospinning of PolyPhe (by NCA polymerization) into the air S3
5. (Figure S5) PolyPhe cast-films S4
6. (Figure S6) Surface roughness of PolyPhe constructs S4
7. (Figure S7) Effect of solvent composition or accelerated voltage on the wettability S5
8. (Figure S8) FT-IR spectra of PolyPhe nonwovens after acidic and basic treatments S5

1. (Figure S1) Electrospinning of PolyPhe into the air

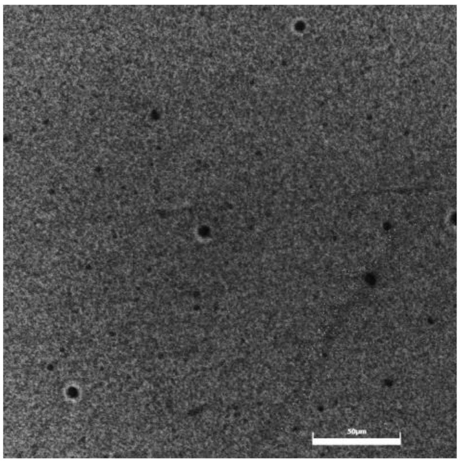

Figure S1. Electrospinning of 32 *w*/*v* % PolyPhe in TFA/$CHCl_3$ (9:1 *v*/*v*) into the air. No fiber formation was observed.

2. (Figure S2) Investigations on length and aspect ratio of the obtained fibers

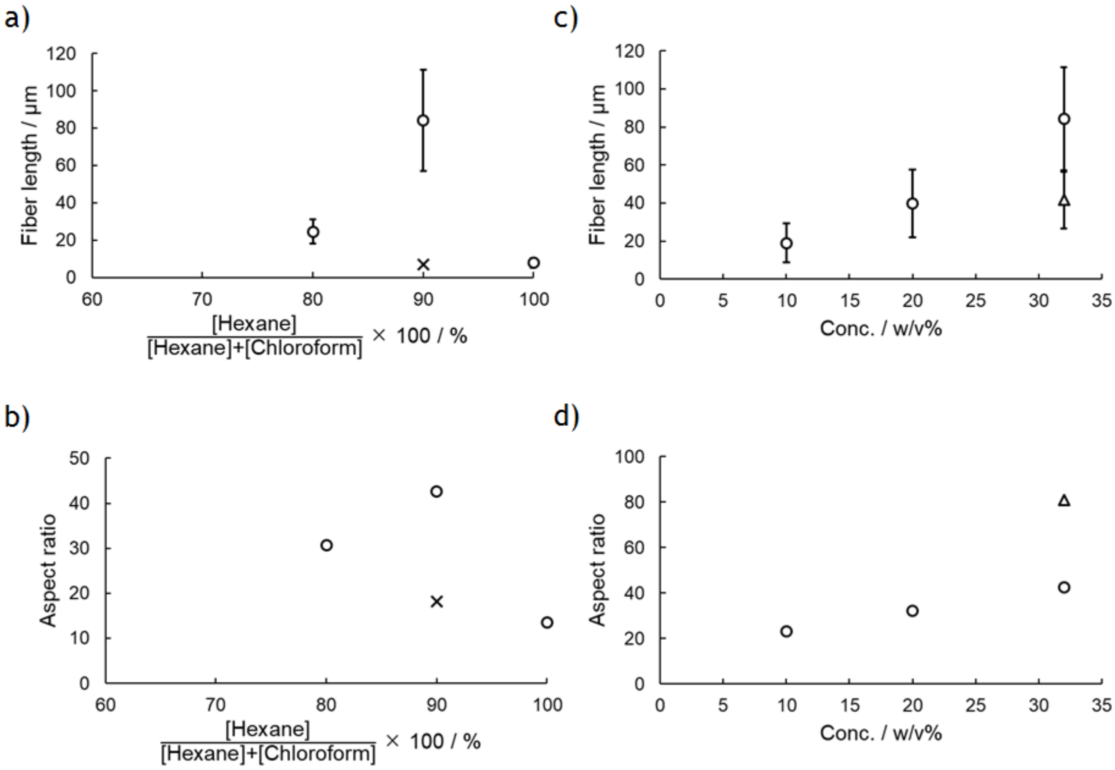

Figure S2. (**a**) Fiber length vs solvent composition; (**b**) aspect ratio vs solvent composition; (**c**) fiber length vs conc.; and (**d**) aspect ratio vs conc. in the electrospinning of PolyPhe into hexane/$CHCl_3$. Data samples shown here are the same as those in **Figure 2** of the main manuscript. The length was calculated from SEM images of three different samples (n = 50). The aspect ratio was calculated as [average fiber length]/[average fiber diameter].

3. (Figure S3) Electrospinning of PolyPhe into cyclohexane/CHCl3 (9/1 v/v)

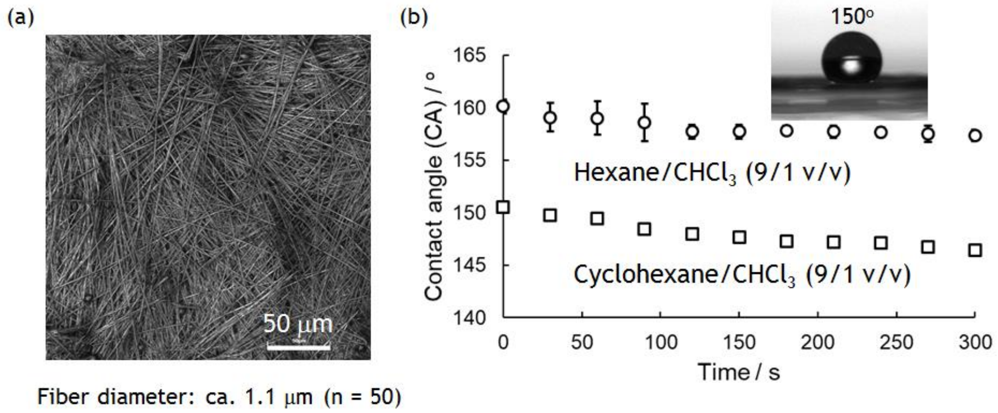

Figure S3. (**a**) Electrospinning of PolyPhe into cyclohexane/$CHCl_3$ (9/1 *v*/*v*). (**b**) Water CA change on the obtained nonwovens (*n* = 3). The inset shows a photograph of a water droplet on the nonwoven. For a comparison, the CA result in the case of hexane/$CHCl_3$ (9/1 *v*/*v*) is also shown.

4. (Figure S4) Electrospinning of PolyPhe (by NCA polymerization) into the air

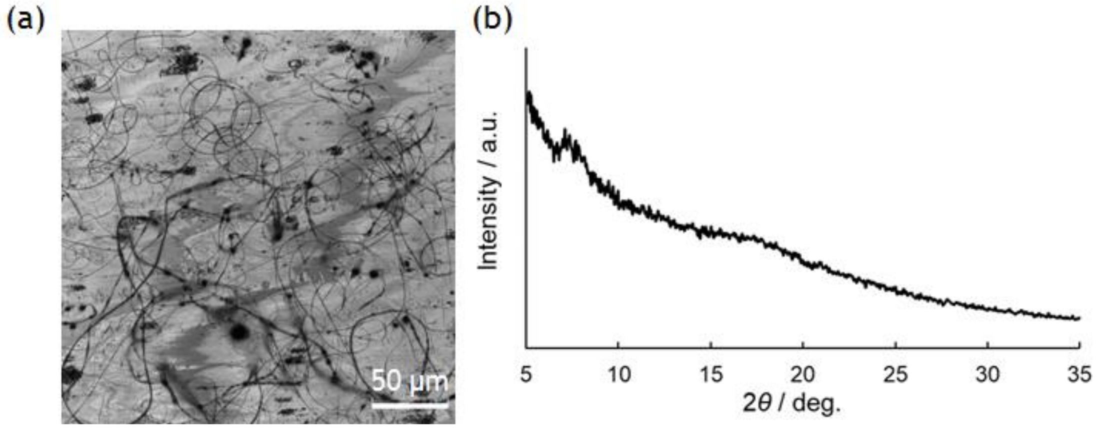

Figure S4. Electrospinning of PolyPhe into the air which was synthesized by polymerization of Phe-NCA in the presence of triphosgene and trimethylamine. PolyPhe was dissolved in $CHCl_3$/TFA (9/1 *v*/*v*) at the concentration of 10 w/v %. Electrospinning was done at the voltage of 30 kV, solution speed was 0.95 mL/h, and collection distance was 10 cm. The molecular weight of the polymer used here was not investigated. (**a**) LM image and (**b**) XRD pattern of the obtained PolyPhe fibers.

5. (Figure S5) PolyPhe cast-films

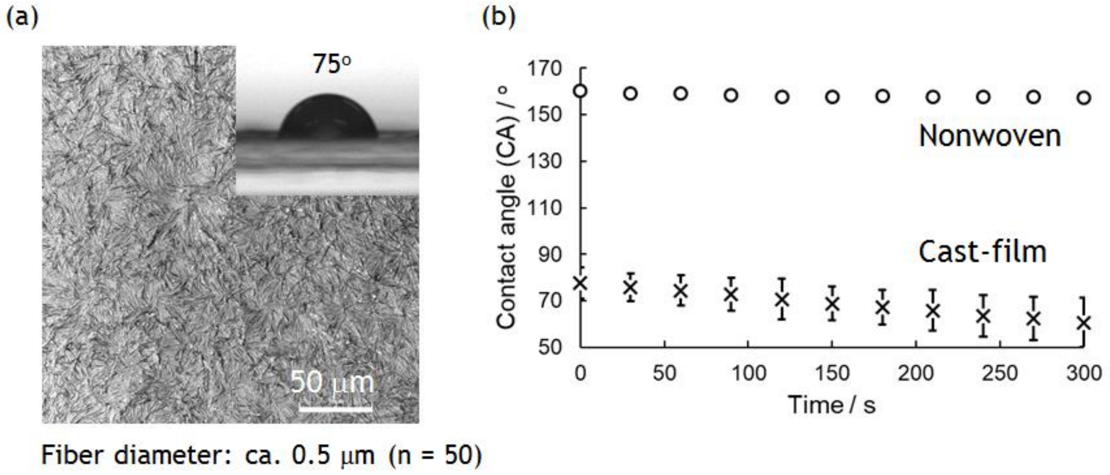

Figure S5. (**a**) LM image of the cast-films prepared by air-drying 32 *w*/*v* % PolyPhe in TFA/$CHCl_3$ (9/1 *v*/*v*) on a glass. The inset shows a photograph of the initial CA of a water droplet on the film. (**b**) Water CA change on the cast-films with time (n = 3). For a comparison, the CA result on the PolyPhe nonwovens is also shown.

6. (Figure S6) Surface roughness of PolyPhe constructs

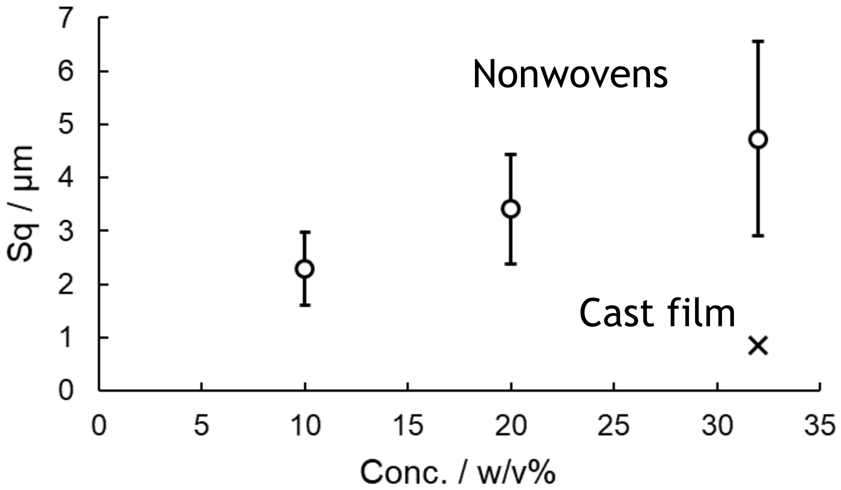

Figure S6. Surface roughness of the PolyPhe nonwovens prepared by electrospinning at different polymer concentrations and the cast-film prepared by air-drying 32 *w*/*v* % PolyPhe (n = 3).

7. (Figure S7) Effect of solvent composition or accelerated voltage on the wettability

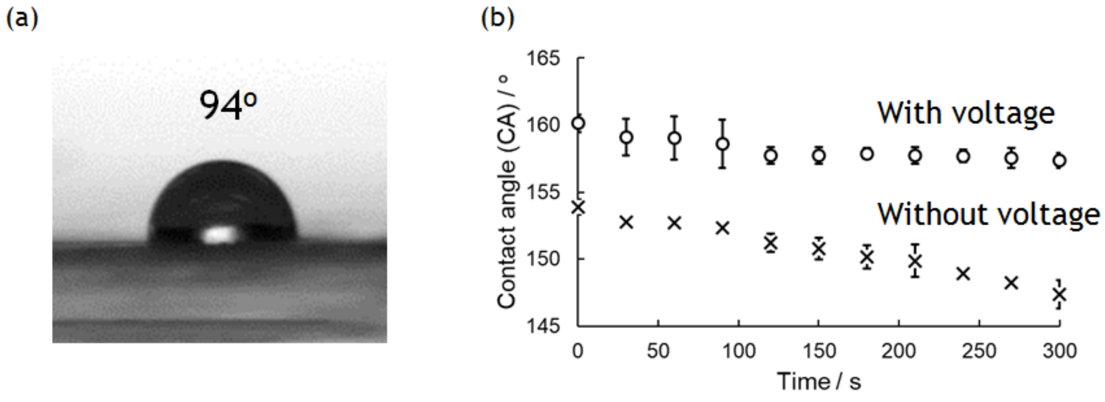

Figure S7. (**a**) A photograph of initial CA of a water droplet on the nonwovens prepared of electrospinning of 32 *w*/*v* % PolyPhe in hexane/$CHCl_3$ (4/1 *v*/*v*). (**b**) CA change of water droplets on the nonwovens prepared by addition of 32 *w*/*v* % PolyPhe into hexane/$CHCl_3$ (9/1 *v*/*v*) under no voltage.

8. (Figure S8) FT-IR spectra of PolyPhe nonwovens after acidic and basic treatments

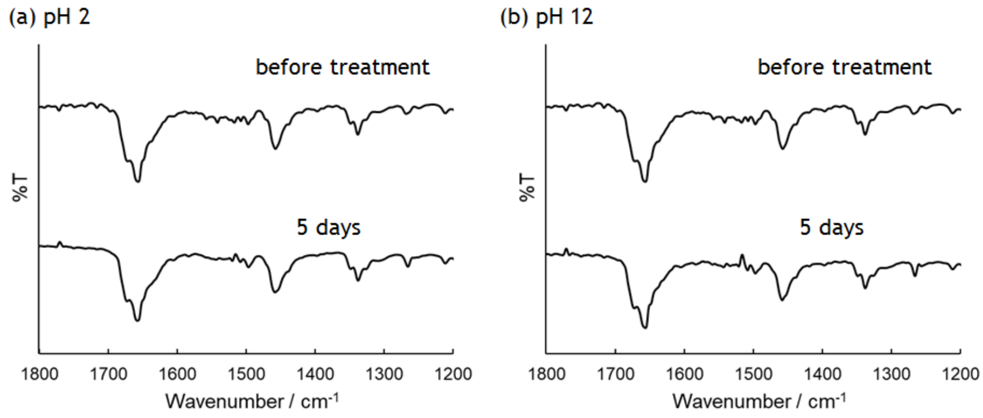

Figure S8. FT-IR spectra of the PolyPhe nonwovens before and after acidic and basic treatments.
